# Evaluation of an onco-diet on body composition and inflammatory status of dogs with mammary tumor—Pilot study

**DOI:** 10.1371/journal.pone.0287797

**Published:** 2023-07-06

**Authors:** Brana S. A. Bonder, Fabio A. Teixeira, Mariana Y. H. Porsani, Lucas A. Gonçales, Julio K. Nagashima, Clair M. de-Oliveira, Julio C. C. Balieiro, Karina Pfrimer, Cristina de O. Massoco, Denise T. Fantoni, Cristiana F. F. Pontieri, Marcio Antonio Brunetto

**Affiliations:** 1 School of Veterinary Medicine and Animal Science, University of São Paulo, São Paulo/Pirassununga, São Paulo, Brazil; 2 Ribeirao Preto Medical School, University of São Paulo, Ribeirão Preto, São Paulo, Brazil; 3 Grandfood Industria e Comercio LTDA, Dourado, São Paulo, Brazil; University of Life Sciences in Lublin, POLAND

## Abstract

A high-protein hypercaloric diet enriched with glutamine and omega-3 polyunsaturated fatty acids was called an onco-diet. The goal was to verify the modulation of the inflammatory response and body composition of female dogs with mammary tumor after mastectomy, during onco-diet consumption, using a randomized, double-blinded, clinical trial. Six bitches (average age of 8.6 years) were allocated into Control Group—diet without glutamine, EPA and DHA supplementation; and six bitches (10.0 years) were allocated into Test—diet enriched with glutamine and omega-3. Serum measurements of TNF-α, IL-6, IL-10, IGF-1, C-reactive protein and determination of body composition were performed at pre- and post-surgical times. Statistical tests were used to compare the nutrient intake and dietary effects on inflammatory variables between the diets. No differences in concentrations of different cytokines (p>0.05) and C-reactive protein (CRP) (p = 0.51) were observed between the groups. The test group had a higher concentration of IGF-1 (p<0.05), higher percentage of muscle mass (p<0.01) and lower body fat (p<0.01), but the difference was present from initial and throughout the study. Onco-diet, enriched with glutamine and omega-3, in the amounts evaluated in this study, was not sufficient to modulate the inflammation and body composition of female dogs with mammary tumors submitted to unilateral mastectomy.

## Introduction

For dogs with cancer, the available recommendations indicate feeding with a high calorie and high protein diet in order to minimize the loss of muscle mass, improve the immune and inflammatory status and, consequently, improve the prognosis [1]. Moreover, there are specific commercial formulas that are enriched with some nutrients purported to be immune-modulating as glutamine, omega-3 fatty acids, arginine, nucleotides, beta-carotene, and/or branched-chain amino acids. These formulas are indicated for humans surgical patients, since it is believed to improve their outcome due to “immunonutrition” effects of these formulas [2].

Glutamine is on the list of possible immunonutrients [3] and its supplementation is associated with improved immune function through better lymphocytic response, maintenance of an adequate intestinal barrier and body musculature. These effects are related to the minimization of oxidative stress due to less glutathione depletion, less cellular death and reduced production of pro-inflammatory cytokines in human patients with cancer and/or submitted to extensive surgical procedures [3–5]. Changes in the expression of enzymes related to glutamine metabolism were recently observed in female dogs with mammary tumors [6]. These findings show that there may be a relationship between tumor growth and glutamine metabolism, which raises the hypothesis that supplementation of this amino acid may be beneficial to cancer patients, including those with mammary neoplasia.

A higher intake of lipids is beneficial because it provides essential fatty acids besides being the most concentrated source of energy among other nutrients, which favors the formulation of hypercaloric diets, which are critical for animals that are in a hypermetabolic state and at risk of weight loss such as dogs with cancer [1, 8]. There are two main families of polyunsaturated fatty acids (PUFA): omega 6 (Ω-6) and omega 3 (Ω-3) and the main fatty acids of these respective families are linoleic acid (18:2n-6), the precursor of arachidonic acid [AA (20:4n-6)]; α-linolenic acid (18:3n-3), which is converted to eicosapentanoic acid [EPA (20:5n-3)] and docosahexaenoic acid [DHA (22:6n-3)] [7, 8]. The PUFAs have several biological actions, among them is serving as substrates for the biosynthesis of bioactive molecules such as prostaglandins, thromboxanes and leukotrienes [8–10]. Many PUFAs constituted of 20 carbons are precursors of eicosanoids; however, AA is considered the main one due to its abundance in the cellular membrane [7].

Eicosanoids modulate the inflammatory response unevenly. The mediators synthesized from AA (prostaglandin E2, prostaglandin I2, prostaglandin D2, prostaglandin F2α, thromboxane A2, leukotriene B4) have greater pro-inflammatory potential capable of inducing vasodilation, bronchoconstriction, platelet aggregation and increased vascular permeability [11]. In contrast, those from the EPA are prostanoids and leukotrienes which are less inflammatory (prostaglandin E3, prostaglandin I3, thromboxane A3, leukotriene) [2]. Thus, it is possible to modulate biological action according to the type of PUFA that will be the primary substrate in the inflammatory cascade, which could be affected by the proportion of dietary Ω-6 and Ω-3 (mainly Ω-3 EPA), as this reflects in the concentration of each PUFA incorporated in cell membranes [11, 12]. The production of cytokines stimulates the proliferation of lymphocytes, mediates the communication between different cell types and controls the immunological responses in order to maintain total body homeostasis [13]. Among all the cytokines, three main inducers of signs of disease stand out: tumor necrosis factor-alpha (TNF-α), interleukin-1 (IL-1) and interleukin-6 (IL-6. The central nervous system functions as a receptor and amplifier of peripheral inflammatory signals and thus commands the responses correlated with acute and chronic disease, important for the body’s defense, such as anorexia, fever, lethargy and protein catabolism. However, these long-term changes may lead to neurodegeneration and cachexia [14]. The action of cytokines occurs mainly in the hypothalamus, as it is a circumventricular organ and does not present a blood-brain barrier [15]. The hypothalamus is the regulating center of appetite, muscle mass and energy homeostasis [16]. It contains the subpopulation of anorexigenic and orexigenic neurons, which decrease and increase appetite, respectively, and can be modulated by cytokines [17]. In addition, the presence of cytokines in the central nervous system activates the corticotropin release factor, one of the key mediators involved in the endocrine stress response. Thus, pro-inflammatory cytokines participate in the neurohormonal imbalance of appetite-regulating signals, induces changes in gene expression, protein synthesis, neuroendocrine signaling, reduce muscle mass [18–20]. Another influential factor for these metabolical changes is the action of inflammatory cytokines in the inhibition of insulin-like growth factor 1 (IGF-1), a potent stimulator of skeletal muscle production [21]. In particular, TNF-α induces resistance to the anabolic effects of IGF-1 [22].

Clinically, weight loss marked as muscle atrophy is considered a prognostic factor for cancer patients. In female dogs with mammary tumor, it was found that the highest gene expression of IL-6 and TNF-α was associated with worst prognoses with an inverse correlation between TNF-α and survival time. On the other hand, greater expression of IL-10 was correlated to better prognoses, which reinforces the use of cytokines as possible prognostic markers [23]. Evidence also suggests that IL-6 is involved in the development of cancer cachexia. In an experimental model with murine colon-26 adenocarcinoma, higher concentrations of IL-6 correlated with cachexia and the use of cytokine neutralizing antibodies attenuated weight loss [24].

Currently, Ω-3 fatty acids have been used in the nutritional support of post-surgical patients to mitigate the exacerbated inflammatory response [7]. A meta-analysis study suggested an association between more complications when using lipid emulsion infusion whose main component was PUFA Ω-6 in critically ill human patients [25]. In veterinary medicine, the benefits of using Ω-3 have been reported in animals with different diseases [9, 26–28]. Despite the limited number of studies about Ω-3 on animals with cancer, there are positive results such as longer survival times of dogs with stage III lymphoma treated with doxorubicin that were supplemented with Ω-3 and arginine [29].

Few studies have effectively evaluated the effect of a “onco-diet” (diet with a profile indicated for cancer patients [1]) on canine inflammatory and immunological markers, hence the goal of this study was to evaluate the effects of a onco-diet (high-protein diet, enriched with glutamine and omega-3 polyunsaturated fatty acids) on the inflammatory status and body composition of female dogs with mammary cancer, submitted to unilateral mastectomy and ovary hysterectomy (OH). The authors hypothesize that this diet can modulate the inflammatory status and result in the maintenance or increase of lean body mass in dogs.

## Materials and methods

This was a double blind randomized clinical trial. All procedures were conducted in accordance with the ethical principles of animal experimentation and approved by the Ethics Committee on the Use of Animals of the School of Veterinary Medicine and Animal Science of the University of São Paulo (protocol n° 6348210316). In addition, all animal owners have signed the informed consent form.

### Animals

Dogs were selected from the routine care of Small Animals Obstetrics and Gynecology Service of the Veterinary Teaching Hospital of the School of Veterinary Medicine and Animal Science, University of São Paulo (SVMAS/USP). Our minimal sample size was estimated as 6 dogs in each group based on previous and similar studies [30] and according to the recommendation of a minimal of 6 animals for metabolic evaluation [31]. The inclusion criteria were uncastrated female dogs with mammary cancer; aged between 1 and 13 years; fed a nutritional complete commercial dry food and not pregnant; for whom there was an indication of mastectomy as a treatment. Exclusion criteria were: other diseases; mammary tumor with clear characteristics of inflammation and ulceration; animals with body condition score greater than or equal to 8 on the 9-point scale [32], ultrasonographic evidence of abdominal metastasis; radiographic evidence of thoracic metastasis; image evidence of enlarged internal lymph nodes; presence of concomitant diseases; animals handled with commercial food indicated for treatment of dermatopathies; presence of pulmonary metastasis; dogs with prescription of fish oil and/or steroid or non-steroidal anti-inflammatory for 6, 2 and 3 weeks, respectively.

The initial screening of the dogs was performed through anamnesis, physical examination and complementary tests—glycemia, complete blood count (CBC); dosage of serum concentrations of creatinine, urea, total proteins, albumin, potassium, triglycerides, cholesterol, evaluation of the serum enzymatic activity of alkaline phosphatase and alanine aminotransferase.

### Diets

Two distinct nutritional complete dry, extruded diets were used in the study (Table 1). The Control diet had a moderate content of protein and fat, with no addition of fish oil (source of EPA and DHA) and no other nutraceuticals (control group). While test food had more protein, fat and calories, in addition to being enriched with glutamine (1.65% DM), EPA (0.7%) and DHA (0.44%), that is, with characteristics of a canine “onco-diet" (test group). Both were formulated in a specific software and extruded at the Grandfood Indústria e Comércio Ltda (Dourado, Brazil). Analyses of dry matter (DM), ash, crude protein and fat content were performed according to AOAC (1995) methods [33] at the Chemistry Laboratory of Grandfood Indústria e Comércio Ltda.

**Table 1:** Nutritional profile of control and test diets used in the study.

	Control diet^1^		Test diet^2^	
Nutrient	% DM	g/1000 kcal	% DM	g/1000 kcal
Protein	25.5	60.4	43.7	91.4
Fat	13.3	31.5	27.3	57.1
Crude fiber	3.3	7.8	2.4	5.0
Ash	8.3	19.7	5.9	12.3
Calcium	1.7	4.0	1.1	2.3
Phosphorus	0.7	1.6	0.8	1.6
Glutamine	0.0	0.0	1.6	3.5
Glutamine + glutamic acid	3.0	7.1	7.5	15.7
Ω-6	2.2	5.3	3.7	7.8
Ω-3	0.3	0.6	1.4	2.9
Ω-6: Ω-3	8.2		2.7	
EPA+DHA	0.0	0.0	1.1	2.3
Metabolizable energy	4219 kcal/kg		4778 kcal/kg	

DM = dry matter; Ω-6 = Omega-6 polyunsaturated fatty; Ω-3 = Omega-3 polyunsaturated fatty; EPA = eicosapentaenoic acid; DHA = docosahexaenoic acid.

^1^Composition: corn (inclusion 47.5%), chicken by-product meal (27.9%), broken rice (8.0%), chicken fat (4.1%), beet pulp (2.5%), rice bran (2.0%), hydrolysed chicken (2.5%), lard (2.0%), dried brewer’s yeast (1.0%), flaxseed (1.0%), salt (0.5%), potassium chloride (0.3%), mineral-vitamin premix (0.4%), bentonite (0.1%), yeast cell wall (0.1%), antioxidant [(BHA and BHT) 0.1%].

^2^Composition: wheat gluten (20.0%), chicken by-product meal (18.9%), broken rice (13.7%), chicken fat (9.35), isolated pork protein (6.0%), lard (6.0%), corn gluten 60 (6.0%), refined fish oil (4.0%), beet pulp (4.0%), whole egg powder (2.5%), dried brewer’s yeast (2.0%), hydrolysed chicken (3.5%), cellulose (1.5%), potassium chloride (1.1%), mineral-vitamin premix (0.7%), mannanoligosaccharides (0.2%), fructo-oligosaccharides (0.2%), taurine (0.1%), yeast cell wall (0.1%), propionic acid (0.1%), yucca extract (0.1%), antioxidant [(BHA) (0.1%)].

### Experimental design

The included animals were randomly divided by drawing lots into two experimental groups to receive either the control food or the test food. The draw was performed prior to the inclusion of animals using Microsoft Excel^®^ software. Diets were delivered to the dog owners, in a double-blinded way and in individual laminated packages that were previously identified with the letters A or B, so that the researchers and owners would not know which treatment each animal was submitted. To ensure the correct delivery of the daily amount of food, each individual package contained the exact daily quantity of feed and was also identified with the date that should be offered to the animal. The daily energy intake was determined in order to provide 95 kcal x (body weight kg)^0.75^ (NRC, 2006) [31] or according to previous caloric intake based on the food quantity information described by the owner during anamnesis. The owners were instructed to store, if any, the daily leftovers. These were weighed and the calculation of the average daily intake of DM, energy and nutrients (protein, fat, fibers, total omega-6, total omega-3 and EPA+DHA) of each experimental group was performed, before and after the surgery, based on the metabolic weight of the dogs (body weight in kg^0.75^).

Each diet was provided for 51 days. The initial consumption occurred 21 days before the surgical procedure (T_-21_) and the end, 30 days after the surgical procedure (T_30_). Samples of 10 mL of blood were collected by venipuncture of the jugular vein after a 12-hour fasting for food and 2-hour fasting for water, at least on T_-21_; on the day of the surgical procedure, before the application of pre-anesthetic medication (T_0A_); in the immediate postoperative period (T_0P_); 1 day (T_1_) and 30 days (T_30_) after surgical intervention.

Dogs were evaluated at the veterinary hospital on days T_-21_; T_0_; T_1_; T_10_ and T_30_. At all evaluation period, the dogs were physically examined and, the amount of food consumed was increased or reduced in 10% if there was a loss or gain of weight corresponding to more than 5%. Each owner completed a follow-up questionnaire for each evaluation period with information about the daily food intake, fecal score [34], urine characteristics (volume, color and odor) and observations regarding other changes that the patient may have presented.

### Surgical procedure

All the animals included were submitted to OH by the three-clamp technique [35] and surgical excision of the mammary chain by unilateral mastectomy, both in a single surgical procedure. In cases where females had tumoral formation in both mammary chains it was decided to remove the one with more advanced neoplastic characteristics. All procedures were performed by the same surgeon and anesthesiologists, who did not have any information about which experimental group each animal belonged.

The drug methadone (0.3 mg/kg) was used as a pre-anesthetic medication, and it was administered for post-surgical analgesia (0.2 mg/kg). Anesthetic induction was performed with propofol (5 mg/kg) and maintenance of the anesthetic plan with isoflurane in 100% oxygen. In the trans-operative, fluid therapy was performed with a lactate ringer solution (5 mL/kg/hour). Fentanyl bolus (3 μg/kg) was administered and kept in continuous infusion during the procedure (0.2 to 0.5 μg/kg/minute). At the end of the surgery, for better analgesia, a sterile flexible catheter was inserted into the surgical wound to allow local application of bupivacaine (1mg/kg) diluted in 0.9% sodium chloride solution to obtain a solution in the ratio of 1:2, immediately and 24 hours after the procedure. The catheter was removed after the second application.

During surgery it was used a multiparametric monitor model Dixtal DX 2020 with echocardiography; pulse oximetry; invasive arterial pressure in podal/metatarsal artery; ventilometry and delta P; capnograph with gas analyzer and transesophageal echocardiography. During the trans-operative period, the patient’s blood loss was evaluated to readjust the fluid infusion.

The patients went to their respective homes in the post-surgical period with a prescription of oral amoxicillin with clavulanate (25 mg/kg) and ranitidine hydrochloride (2 mg/kg), both twice a day for seven days and tramadol hydrochloride (5 mg/kg) three times a day for five days.

### Laboratory analysis

For cytokines, CRP and IGF-1 serum concentration dosages, 3 mL of blood were collected in a tube without anticoagulant. The samples were then centrifuged for 10 minutes at 1000xg, the serum aliquots obtained were immediately stored in freezer cryotubes at -80°C and were analyzed at one time. These evaluations were performed in the LEAC technical laboratory—São Paulo, SP—Brazil.

#### Cytokines IL-10, IL-6 and TNF-α

Serum cytokines were measured at T_-21_, T_0A_, T_0P_, T_1_, T_10_ and T_30_. The quantifications were performed through the MILLIPLEX MAP Canine Cytokines panel (CCYTOMAG-90K). The samples and patterns were incubated in a microsphere coated with the specific antibody. After an analyte from the test sample was captured, a biotinylated detection antibody was introduced. This mixture was incubated with Streptavidin—PE conjugate to complete the surface reaction of the microsphere. The samples were analyzed on the Luminex 200—Software x Ponent/Analyst version 4 (Luminex 200, Luminex Corporation, St. Charles, Missouri, USA).

#### C-reactive protein (CRP)

Serum CRP was measured at T_-21_, T_0P_ and T_1_ times and was evaluated by the Elisa C-reactive Canine Protein (CYT296) kit. Control and samples were incubated in microtiter wells coated with polyclonal anti-CRP canine antibody. After washing, the horseradish peroxidase (HRP) labelled polyclonal anti-canine CRP antibody was added to the wells and incubated with the immobilized antibody-CRP complex. After further washing, HRP-conjugated antibody reacted with substrate and tetramethylbenzidine, which was stopped by the addition of acid solution. The absorbance was read by 450 nm wavelength spectrometry. The reading was performed in the Stat Fax Reader model 2100 (Awareness Technology, Palm City, Florida, USA) and the calculations in the MultiCalc program.

#### IGF-1

Measurements were performed in T_-21_, T_0P_ and T_1_ times using the enzyme-linked immunoassay kit for IGF-1 (SEA050Ca). The samples were added to the wells of the microtiter plate, which were coated with IGF-specific antibody with a biotin-specific IGF-1-conjugated antibody. Then, HRP-conjugated avidin was added to each microplate cavity, with subsequent incubation. Tetramethylbenzidine (TMB) substrate solution was placed in wells containing IGF-1, biotin-conjugated antibody and enzyme-conjugated avidin showed color change. The enzyme-substrate reaction was terminated by the addition of sulfuric acid solution and the color change was measured by a spectrophotometer at a wavelength of 450 nm ± 10 nm. The reading was performed in the Stat Fax Reader model 2100 (Awareness Technology, Palm City, Florida, USA) and the calculations in the MultiCalc program.

#### Lymphoproliferation

Lymphoproliferation was performed in the T_-21_, T_0P_ and T_30_ times to verify basal, immediately postsurgical and late postsurgical moment. The sterile technique was used for the isolation of mononuclear peripheral cells from 1 mL of heparinized blood. Blood sample was transferred to Falcon tube and 5 mL of lysis buffer added and centrifuged at 400xg for 5 minutes. Then the cells were washed with 10 mL of PBS (phosphate buffered saline) and re-centrifuged. Then 900 μL of bovine fetal serum and 100 μL of dimethyl sulfoxide were added and the content was transferred to a cryogenic tube, which was stored in a freezer -80°C for 24 hours and then stored in liquid nitrogen until analysis. The cells were prepared in duplicate, with and without marking. For in vitro lymphocyte marking, a protocol according to Quah, Warren and Parrish (2007) was used.

Lymphocyte labelling was performed using carboxyfluorescein diacetate succinimidyl ester (CFSE) and differentiation in accordance with concentration of cells after resuspension. Cells were incubated at room temperature protected from light for 5 min. Then, washed three times with 10 mL PBS and 5% foetal bovine serum and centrifuged at 300 g for 5 min. Stimulation of proliferation was performed in a 96-well plate containing in each well 1 × 10^5^ cells in 100 μL of DMEM (100 μg/mL penicillin and streptomycin, 2 mm glutamine, 0.1 mm 2-mercaptoethanol, and 10% inactivated foetal bovine serum) and 100 μL of diluted in DMEM. These were cultured for 48 hr and were analysed at the Laboratory of Pharmacology and Toxicology of Department of Pathology (SVMAS/USP) [30].

#### Body composition

Body composition was determined at T_-21_, T_10_ and T_30_ by the deuterium isotope dilution method. A subcutaneous 1mL was inoculated per kg of body weight of sterile 10% deuterium solution (2H_2_O_4_). Blood samples were taken 10 minutes before and 2 hours after the administration. Serum was extracted from these and disposed in plastic collecting tubes, with screw cap and sealed with parafilm stored at -20ºC. The serum deuterium concentration analyses were performed at Mass Spectrometry Laboratory of the Ribeirao Preto Medical School, University of Sao Paulo. Percentages of lean mass and fat mass were estimated by the difference of body water, total lean mass and percentage of fat mass following the methodology described by Ferrier et al. (2001) [36] and adapted by Brunetto et al. (2011) [37]. Results are expressed as percentage of body weight as muscle mass or adipose tissue.

### Statistical analysis

The statistical analysis was performed using the PROC GLIMMIX procedure of the Statistical Analysis System, version 9.3 (SAS, 1995). For the analysis of TNF-α, IL-6 and IL-10 concentrations, the statistical model contemplated the fixed effects of diet (Control vs. Test), evaluation time (T_-21_, T_0A_, T_0P_, T_10_ e T_30_), interaction diet x time, besides the random effects of animal and residue. It was considered that the evaluations were performed on the same animals, characterizing a structure of repeated measures in the same experimental units. For these variables (TNF-α, IL-6 and IL-10) the methodology of generalized linear models was used, assuming Poisson residual distribution and logarithmic link function. For the analysis of the other variables, which presented normal behavior and homogeneity of conditional residues, the statistical model contemplated the same fixed effects (diet, time and diet x time interaction) and random effects (animal and residue) previously described, and in this case, the general mixed linear method was used, through the PROC MIXED procedure of SAS. For these variables it was also considered that the evaluations were performed on the same animals, characterizing a structure of repeated measures in the same experimental units. In case of significant effects on the analysis of variance (ANOVA), the means of least squares were compared by Tukey’s test, using the option AJUSTED = TUKEY, for both procedures mentioned. In order to evaluate the possible relationship between the different variables, Pearson’s Momentum-Product correlations were also performed through the SAS PROC CORR procedure.

## Results

During the selection process of dogs, 14 bitches diagnosed with mammary tumor met the inclusion criteria and were selected, however one was excluded due to unauthorized use of corticoid during the pre-surgical period and one animal was euthanized by decision of the owners during the follow-up period, due to the rapid evolution of the clinical onset associated with cancer. Twelve dogs completed the study. No animal had any change in lymphonode status assessed on physical examination. Control group was composed by two Dachshunds, one Cocker spaniel, one Labrador retriever, one Rottweiler and one mixed breed dog with a mean age of 10.0 (range 6.0 to 13.0) years old. Tumors were classified [38] as adenoma-simple; carcinoma complex type (ductal carcinoma and intraductal papillary carcinoma); carcinoma complex type grade I and carcinoma mixed grade I; carcinoma complex type (ductal carcinoma and Intraductal papillary carcinoma); other sarcoma; carcinoma in situ.

Test group was composed by one Beagle, one Dachshund, one German shepherd, one Poodle and two mixed breed dogs, with a mean age of 8.6 (range 5.0 to 13.0) years. No age differences (*p* = 0.49) between the groups were detected. Tumors were classified as benign mixed tumor and adenoma-simple; adenoma-simple and complex adenoma; benign mixed tumor, carcinoma-simple tubular grade I and carcinoma-simple cystic-papillary grade I-II; carcinoma complex grade I; carcinoma complex type (ductal carcinoma); and carcinoma in situ.

There was no initial difference (*p >* 0.05) between the groups regarding body condition score (BCS), muscle mass score (MMS), serum cytokine concentration, CRP, IGF-1 and lymphoproliferation (Table 2). Over time, the comparison between the two groups showed no variation in body weight (*p* = 0.99), BCS (*p* = 0.95), and MMS (*p* = 0.56) during the period evaluated. Of these 12 animals, seven presented neoplasia in both mammary chains, from which it was decided to remove the chain with more advanced neoplastic characteristics. Three animals were randomly allocated to the control group and four animals were randomly allocated to the test group.

**Table 2:** Initial (T_-21_) body weight, body condition score, muscle mass score and inflammatory parameters of groups A and B (mean ± standard error).

Variable	Control group	Test group	p-value
Body weight (kg)	17.93 ± 5.17	16.56 ± 5.17	0.259
BCS (9-point scale)	5.5 ± 0.47	4.5 ± 0.47	0.160
MMS (0 to 3 points scale)	2.66 ± 0.21	2.33 ± 0.21	0.290
TNF-α (pg/mL)	31.99 ± 21.16	149.79 ± 98.72	0.247
IL-6 (pg/mL)	81.97 ± 41.31	336.05 ± 168.97	0.177
IL-10 (pg/mL)	29.83 ± 14.01	45.75 ± 21.44	0.517
CRP (ng/mL)	0.40 ± 0.01	0.40 ± 0.01	0.567
IGF-1 (ng/mL)	1.32 ± 2.25	7.65 ± 2.25	0.702
Lymphoproliferation (%)	44.16 ± 3.45	42.66 ± 3.45	0.793

BCS = body condition score; MMS = muscle mass score; TNF-α = tumoral necrosis factor-alpha; IL = interleukin; CRP = C-reactive protein; IGF-1 = insulin-like growth factor 1.

Food intake as grams of nutrients per Kg of metabolic weight (kg^0.75^) was analyzed and compared between groups. In each group there was no variation in nutritional intake over time. Although there was no difference in dry matter (*p* = 0,17), fiber (*p* = 0.37) and energy (*p* = 0.06) intake between the groups, as expected, the test group ingested more protein (13.3 *vs* 6.3 g/kg^0.75^; *p* = 0.0011), fat (8.3 *vs 3*.*3* g/kg^0.75^; *p* = 0.0004), omega-6 (1.1 *vs* 0.5 g/kg^0.75^; *p* = 0.0013) and omega 3 (401.0 *vs* 64.2 g/kg^0.75^; *p* <0.0001), compared to the dogs in the control group. In the test group, the average glutamine intake was 496 ± 0.182 mg/kg^0.75^ and EPA+DHA was 0.326 ± 0.120 g/kg^0.75^; while in the control group there was no consumption of these nutrients.

Regarding the inflammatory parameters (Table 3), no dietary effect was observed in the CRP and lymphoproliferation concentrations over time, with no differences between treatments. The diet had an effect in the IGF-1 concentrations when time was not considered, but the actual diet does not appear to have caused the difference as the difference was present from initial and throughout the study. Regarding cytokines, there was a significant difference in the serum concentration of IL-6, TNF-α and IL-10 without diet effect, but with a high variability in cytokine concentrations at each time period.

**Table 3:** Mean concentrations of inflammation-related variables and body composition results at different times in female dogs receiving the control diet and the test diet at pre and post-surgical times (mean ± standard error).

Variables	Diet	Pre-surgical		Post-surgical				p-value		
		T_-21_	T_0A_	T_0P_	T_1_	T_10_	T_30_	Diet	Time	Diet x Time
TNF-α (pg/mL)	CONTROL	31.99^b^ ± 21.16	69.34^a^ ± 45.81	22.33^c^ ± 14.78	23.46^c^ ± 15.53	18.65^d^ ±12.35	23.52^c^ ±15.57	0.256	<0.001	<0.001
	TEST	149.79^a^ ± 98.72	92.33^b^ ± 60.88	85.45^c^ ± 56.34	94.81^b^ ± 62.51	61.89^d^ ± 40.82	45.64^e^ ± 30.11			
IL-6 (pg/mL)	CONTROL	81.97^a^ ± 41.31	19.46^d^ ± 9.88	72.89^b^ ± 36.74	69.84^b^ ± 35.21	73.66^b^ ± 37.13	51.59^c^ ± 26.03	0.177	<0.001	<0.001
	TEST	336.05^a^ ± 168.97	215.51^c^ ± 108.38	195.07^d^ ± 98.11	226.28^b^ ± 113.79	74.86^f^ ± 37.69	111.73^e^ ± 56.22			
IL-10 (pg/mL)	CONTROL	29.84^b^ ± 14.02	29.41^b^ ± 13.82	24.10^c^ ± 11.34	17.42^d^ ± 8.22	32.06^b^ ± 15.05	40.69^a^ ± 19.08	0.516	<0.001	<0.001
	TEST	45.75^b^ ± 21.45	37.86^cd^ ± 17.77	42.09^bc^ ± 19.74	36.02^d^ ± 16.91	64.70^a^ ± 30.28	36.76^cd^ ± 17.25			
CRP (ng/mL)	CONTROL	0.40 ± 0.01	-	0.42 ± 0.01	0.41 ± 0.01	-	-	0.513	0.615	0.568
	TEST	0.41 ± 0.01	-	0.41 ± 0.01	0.42 ± 0.01	-	-			
IGF-1 (ng/mL)	CONTROL	1.32^B^ ± 2.26	-	2.08^B^ ± 2.26	1.66^B^ ± 2.26	-	-	0.035	0.878	0.702
	TEST	7.65^A^ ± 2.26	-	4.59^A^ ± 2.26	6.23^A^ ± 2.26	-	-			
Lymphop. (%)	CONTROL	44.17 ± 3.50	-	-	-	46.67 ± 3.50	43.70 ± 2.95	0.792	0.589	0.982
	TEST	42.67 ± 3.50	-	-	-	46.42 ± 3.50	43.17 ± 2.95			
MM (%)	CONTROL	65.17^A^ ± 3.44	-	-	-	68.23^A^ ± 3.76	71.03^A^ ± 3.44	0.005	0.815	0.465
	TEST	80.19^B^ ± 3.44	-	-	-	76.56 ± 3.44	77.88 ± 3.44			
Body fat (%)	CONTROL	34.82^A^ ± 3.43	-	-	-	31.76^A^ ± 3.76	28.96^A^ ± 3.44	0.005	0.815	0.465
	TEST	19.79^B^ ± 3.43	-	-	-	23.43^B^ ± 3.44	22.10^B^ ± 3.44			

Means in the same line and followed by the same lower case letter do not differ at the 5% probability level by Tukey Test. Means in the same column and followed by the same capital letter do not differ at the 5% probability level by Tukey Test. TNF-α = alpha tumor necrosis factor; IL = interleukin; CRP = C-reactive protein; IGF-1 = insulin-like growth factor 1; Lymphop. = Lymphoproliferation; MM = muscle mass; T_0A_ = moment imediatelly presurgical; T_0P_ = moment postsurgical.

Albeit no differences in BCS and MMS between groups at T_-21_, the control group presented greater percentage of body fat than the test group and consequently less muscular mass and this was remained throughout the study. The diet had no effect in the percentage of fat and muscle mass when comparing each time point individually.

When evaluating the animals individually, it was identified that there was an animal in the test group that presented an outlying serum concentration of cytokines. In order to account for its influence on the results, the outlier was removed, and the analysis redone, however the results remained unchanged. Individually results may be observed in Tables 4 and 5.

**Table 4:** Individually concentrations of inflammation-related variables and body composition variables at different times in female dogs receiving the control diet at pre- and post-surgical times.

ID	Variable	Pre-surgical		Post-surgical			
		T_-21_	T_0A_	T_0P_	T_1_	T_10_	T_30_
**1**	TNF-α (pg/mL)	2.59	2.59	2.59	2.59	2.59	133
	IL-6 (pg/mL)	9.02	13.36	27.82	28.93	9.02	120
	IL-10 (pg/mL)	5.33	6.45	16.77	5.7	3.62	8.06
	CRP (ng/mL)	0.396	-	0.395	0.399	-	-
	IGF-1 (ng/mL)	0.651	-	0.367	1.210	-	-
	Lymphoproliferation (%)	39	-	42	-	-	45
	Muscle mass (%)	75.74	-	-	-	73.99	76.54
	Body fat (%)	24.25	-	-	-	26.00	23.45
**2**	TNF-α (pg/mL)	166.0	131.0	127.0	98.9	13.9	140.0
	IL-6 (pg/mL)	102.0	83.2	133.0	130.0	32.3	122.0
	IL-10 (pg/mL)	14.15	14.66	23.56	9.81	32.16	13.64
	CRP (ng/mL)	0.426	-	0.416	0.413	-	-
	IGF-1 (ng/mL)	0.878	-	0.555	1.400	-	-
	Lymphoproliferation (%)	46	-	45	-	-	43
	Muscle mass (%)	53.71	-	-	-	54.80	57.46
	Body fat (%)	46.28	-	-	-	45.19	42.53
**3**	TNF-α (pg/mL)	2.59	2.59	2.59	2.59	2.59	3.35
	IL-6 (pg/mL)	14.47	17.24	22.81	25.60	12.27	20.02
	IL-10 (pg/mL)	9.81	27.17	9.81	6.45	5.33	8.92
	CRP (ng/mL)	0.382	-	0.415	0.404	-	-
	IGF-1 (ng/mL)	0.898	-	2.100	1.540	-	-
	Lymphoproliferation (%)	51	-	50	-	-	43
	Muscle mass (%)	56.79	-	-	-	99.06	79.29
	Body fat (%)	43.20	-	-	-	0.93	20.7
**4**	TNF-α (pg/mL)	20.08	10.57	4.76	3.65	13.7	13.91
	IL-6 (pg/mL)	44.30	44.30	27.82	28.93	37.21	36.66
	IL-10 (pg/mL)	214	240	136	90.13	195	374
	CRP (ng/mL)	0.421	-	0.411	0.395	-	-
	IGF-1 (ng/mL)	3.227	-	6.740	2.755	-	-
	Lymphoproliferation (%)	47	-	49	-	-	31
	Muscle mass (%)	68.93	-	-	-	69.56	70.14
	Body fat (%)	31.06	-	-	-	30.43	29.85
**5**	TNF-α (pg/mL)	353	1091	237	274	300	84.9
	IL-6 (pg/mL)	675	3.35	528	482	703	200
	IL-10 (pg/mL)	91.63	16.77	75.27	67.97	124.00	60.76
	CRP (ng/mL)	0.384	-	0.406	0.405	-	-
	IGF-1 (ng/mL)	2.255	-	2.139	2.743	-	-
	Lymphoproliferation (%)	45	-	44	-	-	52
	Muscle mass (%)	63.24	-	-	-	70.36	68.91
	Body fat (%)	36.75	-	-	-	29.63	31.08
**6**	TNF-α (pg/mL)	33.43	14.34	29.24	41.85	3.96	49.57
	IL-6 (pg/mL)	53.45	51.84	59.29	69.78	13.36	66.65
	IL-10 (pg/mL)	17.85	42.66	23.56	25.95	18.96	15.70
	CRP (ng/mL)	0.403	-	0.455	0.415	-	-
	IGF-1 (ng/mL)	0.028	-	0.603	0.294	-	-
	Lymphoproliferation (%)	37	-	50	-	-	48
	Muscle mass (%)	72.58	-	-	-	72.43	73.85
	Body fat (%)	27.41	-	-	-	27.56	26.14

ID = animal identification; TNF-α = alpha tumor necrosis factor; IL = interleukin; CRP = C-reactive protein; IGF-1 = insulin-like growth factor 1; T_0A_ = moment imediatelly presurgical; T_0P_ = moment postsurgical.

**Table 5:** Individually concentrations of inflammation-related variables and body composition variables at different times in female dogs receiving the test diet at pre- and post-surgical times.

ID	Variable	Pre-surgical		Post-surgical			
		T_-21_	T_0A_	T_0P_	T_1_	T_10_	T_30_
**7**	TNF-α (pg/mL)	193	375	201	229	254	2.59
	IL-6 (pg/mL)	324	672	303	313	211	10.63
	IL-10 (pg/mL)	38.66	46.74	31.53	34.73	287	3.62
	CRP (ng/mL)	0.4		0.427	0.46		
	IGF-1 (ng/mL)	0.564	-	0.093	0.135	-	-
	Lymphoproliferation (%)	35	-	47	-	-	47
	Muscle mass (%)	79.27	-	-	-	80.92	92.50
	Body fat (%)	20.72	-	-	-	19.07	7.49
**8**	TNF-α (pg/mL)	26.93	21.89	18.29	18.29	6.5	5.27
	IL-6 (pg/mL)	79.07	65.6	59.82	91.29	31.7	23.37
	IL-10 (pg/mL)	12.64	12.64	19.52	11.67	13.64	14.66
	CRP (ng/mL)	0.423	-	0.438	0.379	-	-
	IGF-1 (ng/mL)	0.768	-	0.404	0.612	-	-
	Lymphoproliferation (%)	68	-	45	-	-	54
	Muscle mass (%)	67.35	-	-	-	66.70	70.63
	Body fat (%)	32.64	-	-	-	33.29	29.33
**9**	TNF-α (pg/mL)	2.92	168.00	100.00	26.69	62.15	2.59
	IL-6 (pg/mL)	18.35	185.00	193.00	72.89	74.96	12.27
	IL-10 (pg/mL)	14.15	7.65	15.70	22.38	6.45	11.20
	CRP (ng/mL)	0.392	-	0.369	0.405	-	-
	IGF-1 (ng/mL)	0.404	-	0.028	0.620	-	-
	Lymphoproliferation (%)	34	-	46	-	-	46
	Muscle mass (%)	76.46	-	-	-	79.19	74.67
	Body fat (%)	23.53	-	-	-	20.80	25.32
**10**	TNF-α (pg/mL)	212.00	4.12	2.59	2.59	3.96	15.21
	IL-6 (pg/mL)	453.00	22.25	17.79	21.7	21.7	337.00
	IL-10 (pg/mL)	111.00	42.66	21.80	30.89	49.50	122.00
	CRP (ng/mL)	0.425	-	0.419	0.413	-	-
	IGF-1 (ng/mL)	24.29	-	10.108	20.1	-	-
	Lymphoproliferation (%)	50	-	61	-	-	34
	Muscle mass (%)	79.89	-	-	-	78.30	75.16
	Body fat (%)	20.10	-	-	-	21.69	24.83
**11**	TNF-α (pg/mL)	18.29	61.22	81.03	13.49	81.26	53.54
	IL-6 (pg/mL)	56.11	94.31	146.00	31.15	118.00	102.00
	IL-10 (pg/mL)	22.38	50.89	46.74	44.02	44.7	44.02
	CRP (ng/mL)	0.406	-	0.386	0.384	-	-
	IGF-1 (ng/mL)	8.173	-	6.686	5.896	-	-
	Lymphoproliferation (%)	34	-	38	-	-	48
	Muscle mass (%)	96.84	-	-	-	86.12	84.37
	Body fat (%)	3.15	-	-	-	13.87	15.62
**12**	TNF-α (pg/mL)	2078	930	1041	1312	638	692
	IL-6 (pg/mL)	4373	2362	2359	3041	724	1278
	IL-10 (pg/mL)	220	186	250	186	191	141
	CRP (ng/mL)	0.400	-	0.427	0.488	-	-
	IGF-1 (ng/mL)	11.724	-	10.209	9.988	-	-
	Lymphoproliferation (%)	35	-	41	-	-	30
	Muscle mass (%)	81.35	-	-	-	68.13	69.96
	Body fat (%)	18.64	-	-	-	31.86	30.03

ID = animal identification; TNF-α = alpha tumor necrosis factor; IL = interleukin; CRP = C-reactive protein; IGF-1 = insulin-like growth factor 1; T_0A_ = moment imediatelly presurgical; T_0P_ = moment postsurgical.

## Discussion

A surgical procedure of mastectomy and OH in female dogs with mammary cancer was used to evaluate the possible use of a therapeutic diet for hypermetabolism conditions—high-protein, enriched with amino acid glutamine and fish oil, source of EPA and DHA PUFAs. Other studies have already utilized post-surgical tissue injury, including neoplasms, to evaluate nutritional effects on inflammation [29, 30, 38, 39]. In our study six uncastrated female dogs with mammary tumor were used in each experimental group (test *vs* control).

Eicosanoids are involved in both pro-inflammatory and anti-inflammatory processes and numerous investigations have evaluated the modulation of the inflammatory response by correlating the proportion of fatty acids intake (Ω-6 and EPA/DHA) in humans and animals under different circumstances [29, 38, 39]. In order to the immunomodulatory effects associated with omega-3 fatty acids occur, it is necessary to consume them regularly. In this study, the higher protein, fat, glutamine and EPA+DHA intakes by the test group were expected to have some effect, since the test diet had about 2.1 times more fat and 1.8 times more protein (in dry matter), while the control diet was absent in glutamine and supplemented with EPA+DHA. Diets with high protein and fat contents and, consequently, low carbohydrate, are indicated for dogs with malignant neoplasia [1]. Dogs with mammary carcinoma had a higher median survival time after consumption of a food with a higher percentage of their total dietary calories derived from protein [40]. Dogs affected by neoplasia can present alterations in the carbohydrate metabolism and are more susceptible to the development of cachexia [41–44]. In this study, we did not evaluate carbohydrate-related metabolites because they were not among the objectives of the research.

Animals with cancer may present changes in body composition due to changes in nutrient metabolism resulting from the presence of the neoplasia itself, the production of cytokines induced by the disease and the surgical procedure; and also by the hormonal action resulting from OH [43, 45]. For inclusion in the study, a complete physical evaluation was performed, which included BCS and MMS, as they are tools routinely used to estimate the fat percentage and muscle mass condition, respectively [46]. Although subjective, the BCS is a simple, fast, non-invasive and low-cost method [47] and the evaluation of the muscle condition is necessary, especially in diseased animals, since weight itself is not a reliable parameter in these cases [48]. Despite the knowledge about the action of pro-inflammatory cytokines in the development of cancer cachexia, few studies have evaluated the body composition of people with neoplasia and no studies on dogs with cancer have been found so far that have evaluated body composition by objective methods. Baracos et al. (2010) [49] observed that, despite only 7.5% of human patients with lung cancer are underweight and 47.4% overweight, in CT images, muscle depletion was found in 46.8% of cases, regardless of body mass score [49]. In animals, studies on body composition tend to be more directed to the evaluation of the effects of age [50, 51], obesity [52, 53] and castration [54–56] on the musculature. In this study, from the beginning, subjects in the control group had more fatty tissue at the expense of lean mass, and the opposite happened in test group, making it difficult to use this data to evaluate the diet. Despite these limitations which caused by the use of subjective methods for body composition evaluation (BCS and MMS) instead of an objective method such as the deuterium isotopes [32, 36, 46, 57], BCS and MMS were chosen to allow double-blind randomization. In addition, differences in body composition were overlooked by BCS, MMS and in the evaluation of the deuterium since signs of muscular mass loss were absent, given that dogs were already sarcopenic or in cachexia. Moreover, this study found a positive correlation between the BCS and MMS scale and the deuterium method (p<0.001), which denotes the possibility of using these tools in veterinary clinical routine. Finally, there was no weight gain after castration, probably due to intensive monitoring with a controlled amount of food, without a change in caloric intake over time, even though changes in body composition still occurred as previously reported in other studies [45].

IGF-1 is identified as a potent protein synthesis inducer in myoblasts and TNF-α is postulated as one of the causes of muscle mass loss under disease conditions [58]. Da *et al*. (2015) showed that female dogs with pyometra who underwent castration had a higher concentration of CRP both pre- and post-surgical procedure, when compared to healthy female dogs undergoing the same process. Moreover, they demonstrated a significant increase in the IGF-1 from the third to the tenth postoperative day, in contrast to the control group, which did not present changes in concentration throughout the study [59]. In this study, the test group showed an increase in serum IGF-1 concentration from T_0P_ to T_1_, while the opposite effect was identified in the control group (Table 3). No specific study was found to report the influence of glutamine, PUFAs, or other diet components on IGF-1 behavior neither in humans or dogs with cancer. Despite a statistical difference in IGF-1 levels between diet-groups was seen, taking into consideration the differences in baseline values it is impossible affirm a diet effect on this variable.

Cytokines can also favor appearance, growth and tumor metastasis [60]. It is reported that interleukins play an important role in cancer as potent modulators of angiogenesis and leukocyte infiltration. It was found that dogs with inflammatory mammary cancer had higher serum concentrations of IL-6 when compared to healthy female dogs and higher concentrations of IL-10 when compared to both healthy female dogs and female dogs affected by non-inflammatory malignant mammary tumors [61]. Estrela-Lima et al. (2013) [62] found that female dogs presenting mammary carcinoma with metastasis expressed high IL-10 production. This information emphasizes the possible immunosuppressive and anti-inflammatory effects of IL-10 [63], such as the modeling of apoptosis and reduction of angiogenesis in cases of regression [64] and as a beneficial prognostic factor in canine mammary cancer [23]. In this study, although, no difference in the IL-10 concentrations was observed, the serum concentrations of the test group were higher at T_0P_, T_1_ and T_10_, which could indicate an improved inflammatory modulation in these patients [63], but the very high variability and low study power make any conclusions difficult to draw.

To alleviate the undesirable effects of the accentuated production of cytokines, the immunostimulant action and the safety of omega-3 fatty acids are described in numerous reports using emulsion containing fish oil in the parenteral nutrition of critically ill human patients [11, 65, 66]. However, our results corroborate the findings of Mayer et al. (2003) [67], in which cytokine production was observed in human patients with sepsis in conventional emulsion parenteral nutrition therapy (with Ω-6) or with a solution with Ω-3 (based on fish oil), for 5 days. Serum concentrations of TNF-α, IL-6, IL-8 and IL-10 did not differ between groups. However, in this case, when ex vivo cytokine secretion by isolation of mononuclear leukocytes and endotoxin-stimulated monocytes was evaluated elsewhere, a 30% reduction in the release of pro-inflammatory cytokines was found during the infusion of solution with Ω-3, in contrast to the increase in these cytokines with the administration of a solution of Ω-6. The authors attributed this difference in results due to the use of different methodologies employed and to other effects that may alter the concentration of cytokines in the circulation [67], which may also have affected the results of our research.

Part of the effects of fish oil were identified in human patients undergoing abdominal surgery after enriched parenteral infusion, in which amplification of acute-phase proteins of the liver measured by molecular biology methods was detected. Although an increase in concentration was observed after the 5-day infusion period, no difference was found [68]. Mayyas et al. (2011) [69] found that the supply of 0.6g of Ω/kg body weight/day for 3 weeks before heart surgery in dogs was effective in reducing the Ω-6:Ω-3 ratio of the heart muscle (134:1 vs 4:1). However, when plasma CRP concentrations were analyzed, the supplemented and control groups presented similar values. In this context, we believe that the differences in the analytical methods may influence the results found in the current study, as neoplasia and surgical excision of both mastectomy and OH affect the blood concentration of CRP [70], however, the concentration of CRP in this study was not influenced by the glutamine-enriched diet and EPA+DHA.

Leblanc et al. (2008) [71] showed that, in healthy dogs, feeding a diet containing 1.75 grams of EPA/kg of food in the DM and 2.2 grams of DHA/kg of food in the DM and a ratio Ω-6:Ω-3 of 3.4:1 for 12 days was associated with lower total activity of IL-6 in the period of 6 hours after stimulation with lipopolysaccharide, compared to dogs that used sunflower oil diet. However, the test diet was not able to result in a difference in TNF-α concentration between the groups, according to the findings in this study. In turn, in dogs with stage III or IV lymphoma, Ogilvie et al. (2000) [29] verified the effect of fatty acid Ω-3 in concentrations of TNF-α and IL-6 in patients undergoing five sessions of chemotherapy (12 weeks of follow-up). Patients consumed an experimental fish oil supplemented diet (Ω-6 = 23 g/kg DM; Ω-3 = 73 g/kg DM; ratio Ω-6:Ω-3 = 0.3:1) or a control diet supplemented with soy oil (Ω-6 = 125 g/kg DM; Ω-3 = 16 g/kg DM; Ω-6:Ω-3 = 7.7:1). The authors did not observe any difference in TNF-α serum concentrations between groups. However, for IL-6, a reduction in concentration was observed in both groups. These results are in accordance with our observations of a lack of fluctuation in the levels of pro-inflammatory cytokines, even if it occurred for a shorter period (7 weeks) and the ratio Ω-6:Ω-3 used in the test diet is higher.

The supplementation with fatty acids Ω-3 can suppress the proliferation and function of lymphocytes, a fact that may be related to the reduction of the synthesis of pro-inflammatory cytokines that promote the proliferation and differentiation of lymphocytes [72–75]. However, such association was not demonstrated in the current study, as there was no correlation between TNF-α (*p* = 0.08) or IL-6 (*p* = 0.08) with the percentage of lymphoproliferation, maybe explained by the small sample size. Contrary to our results, LeBlanc (2005) [75] which evaluated three distinct supplements in healthy dogs (group 1: Ω-6 total = 16.6 g/d; Ω-3 total = 0.69 g/d; EPA < 0.1 g/d; DHA < 0.1 g/d; Ω-6:Ω-3 = 23.98 vs group 2: Ω-6 total = 10.2 g/d; Ω-3 total = 2.98 g/d; EPA = 0.75 g/d; DHA = 0.15 g/d; Ω-6:Ω-3 = 3.4 vs group 3: group 2 diet + vitamin E = 196.6 IU/d), found reduction in the percentage of lymphoproliferation when analyzed by flow cytometry after twelve weeks, in all groups. However, when these results were expressed in peak numbers, the group treated with the Ω-3-enriched diet had reduction in proliferation in the twelfth week.

While no studies have been found in the literature to allow discussion of glutamine supplementation to dogs with tumor, in human medicine and laboratory animals, studies with oral and parenteral glutamine supplementation are controversial due to the wide use of glutamine by tumor cells and some concerns about whether glutamine supplementation would not aid tumor growth. Although, there is lower concentration of glutathione in natural killer cells of patients with tumor and the this supplementation would be a way to restore glutathione concentration and consequently improve the response against tumor cells [76–79]. The other possible benefits listed would be a reduction in the blood concentration of inflammatory cytokines and CA in patients with cancer and/or submitted to surgical procedures; the better response of the immune system; minimization of weight loss, correction of protein metabolism and better intestinal health with less possibility of bacterial translocation between the intestinal lumen and the bloodstream during radiotherapies in humans [5, 76, 77]. In veterinary medicine, the only study found was performed with cats, who had intestinal injury induced by the administration of chemotherapy methotrexate, and there was no difference between the group supplemented with glutamine and the group that received the diet control with purified amino acids [80].

Studies in other species present controversial results such as no modification in the blood concentration of inflammatory markers, but with the intensification of the response of cells of the immune system such as lymphocytes [4, 81], which can be seen as a negative result given the possible stimulus to the production of inflammatory cytokines [82, 83], but could be interesting for situations of combat to serious diseases that require a wide inflammatory and immunological response [84]. In addition, a correlation between increased glutamine supplementation and mortality of critically ill humans patients had already been observed [85]. Thus, when studies in human medicine are systematically analyzed, the indication of the use of glutamine as an immunonutrient is reduced [86].

Although it is a routine surgical procedure in veterinary medicine, castration can generate complications and elevations in inflammatory markers [87–89]. For the mastectomy, it is expected to cause even more inflammation, since it is an even more aggressive surgery. However, in the present study no increase in serum concentration of inflammatory markers—cytokines and CA—was observed after surgical procedure, which denotes the possible unfeasibility of this procedure as a model for evaluation of an onco-diet that could be indicated to patients in more severe situations. In a study that evaluated the use of enteral nutrition enriched with glutamine and omega-3 to human patients with gastric carcinoma submitted to surgery, there was an attenuation of the inflammatory and immunological response, compared to the non-supplemented group, through analysis of other markers of nutritional status such as pre-albumin and transferrin and, immunological markers such as the count of different cell types such as CD4, immunoglobulin dosage and CD4/CD8 cell ratio [90]. The absence of these markers in our study made it impossible to further evaluate the immunological, inflammatory and nutritional response of our patients, which could lead to different conclusions.

Other limitations should be cited in this study. This study is a "pilot study” because 6 dogs/group is a small sample size and it reduces the power analyses, despite being in accordance with the recommendations of the NRC (2006) [31], for the evaluation of nutritional and metabolic variables of dogs. So, new studies are necessary to reinforce or refute our data. Although, to minimize possible biases in the current study, we used rigorous inclusion criteria, to perform it as a double-blind and randomized clinical trial. However, as it was a clinical trial and we allocated randomly, we had a histopathological classification [91] of tumors only after surgery. The characterizations as benign or malign tumors were similar between groups, and we believed that it did not affect the results. As a financial limitation, we could not measure all laboratory parameters in all experimental moments. Despite these limitations, this is the first study that evaluated the effects of a high-protein onco-diet with glutamine, EPA and DHA in a clinical trial about dogs with mammary neoplasia.

## Conclusions

According to the results obtained in this pilot study for these twelve animals, under the experimental protocol used, hyperprotein food containing 1.4% dry matter (DM) of EPA and DHA, at a 2.7:1 ratio of Ω-6:Ω-3, and 1.65% glutamine was not able to modulate the inflammatory response or change body composition of female dogs with mammary cancer subjected to mastectomy and castration.
